# Three Cases of Puerperal Peritonitis

**Published:** 1849-08

**Authors:** L. M. Lawrence

**Affiliations:** Nashville, Tenn., Resident Pupil at the Bellevue Hospital, New York


Art. II.—*Three Cases of Puerperal Peritonitis.* Reported by L. M. Lawrence, M. D., of Nashville, Tenn., Resident Pupil at the Bellevue Hospital, New York.

*Case.* I.—M. W. aetat. 35 years, native of Ireland, was delivered at 10 o'clock, A. M., on the 21st of December, of her third child. The labor was natural and easy, lasting eight hours.

22nd, 8 A. M. Lochial discharge is going on well.—Pulse 110; mind uneasy; bowels not moved since delivery. Ordered ol. ricin. ℥ iss. which not being retained, gave magnes. sulphat. ℥ j.; operated well.

2 P. M. Has had a violent chill, followed by hot skin, profuse perspiration, and partial secretion of milk; pulse full, 140; occasional vomiting and delirium. Tenderness on pressure in right iliac region. Prescribed,

℞. Hydrarg. submur. gr. x.
Camphor, gr. v.
Pulv. opii, gr. j.
M.

The vomiting was relieved by the free use of ice.

6 P. M. Vomiting returned. Abdomen slightly tympanitic; tenderness in right iliac much increased; great mental uneasiness; pulse 130 and small. Bled her to approaching syncope; pulse fell to 120, and increased in volume.

9 P. M. Patient more tranquil; vomiting has ceased, but intense pain still produced by pressure. Calomel, camphor, etc. repeated; hop fomentation to abdomen.

23d, 9 A. M. Rested pretty well latter part of the night, and is more quiet than on yesterday; had one small alvine discharge; abdomen more tympanitic, and pain in the iliac region greater; pulse 130. Lochia have ceased. Ordered ol. ricin. ℥ j., which was rejected by the stomach; then gave of calomel gr. v., camphor gr. ij., pulv. opi. gr. ss. M.; to be repeated every two hours.

2 P. M. Pulse 120; general condition unimproved.—Directed six leeches to hypogastrium, to be followed by warm poultices. Cal., camph., etc. continued.

5 P. M. Bowels freely moved by an enema of oil and spts. turpentine. A blister 12 by 12 inches to abdomen, to be dressed with mercurial ointment.

24th, 9 A. M. Patient much worse; rested badly; pulse very frequent and feeble; constantly vomiting a greenish yellow matter.

Died at 3 P. M.

*Autopsy.*—Intestines distended with flatus. Peritoneum congested and inflamed in a high degree throughout its entire extent, most so in the right iliac region. A slight effusion of serum in the peritoneal cavity; neither lymph or pus to be anywhere detected. Uterus healthy, save a slight inflammation of the veins. Other viscera unaltered.

*Case* II.—Josephine A——, aet. 22 years, native of Ireland, of intemperate habits, was delivered at 2 A. M. on the 22d of December, after an easy and natural labor, which lasted six hours, of her first child. The patient manifested great anxiety of mind, both during and after labor.

11 A. M. Doing well; being apprehensive of peritonitis I ordered a dose of oil, to be followed, at intervals of an hour, by a powder of calomel gr. v., camphor gr. ij., pulv. opii gr. j.

7 P. M. Bowels moved freely; lochia normal.

23d, 2 P. M. Had a slight chill, followed by high fever, perspiration and a slight secretion of milk. Powders continued, decreasing the dose one-half.

6 P. M. Tenderness on pressure in right iliac region. Pulse tense, 120. Sat patient up in bed, bled her to approaching syncope, taking about twenty ounces of blood. Pulse immediately fell to 110, and became fuller.

24th, 9 A. M. Patient rested well; had been troubled with slight vomiting, which readily yielded to the use of ice. Abdomen somewhat tympanitic; tenderness increased; lochial discharge less copious; face anxious; constant jactitation. Same treatment.

6 P. M. Tenderness and tympanitis augmented; tongue coated; pulse 130, and tense. Venesection to sixteen ounces was practiced, and under it the pulse became more natural.

25th, 9 A. M. Patient passed a tolerably quiet night. No alvine dejection; complains of still greater pain in the abdomen; occasionally vomits, and the lochia have been arrested. Directed an enema of oil and turpentine; bran fomentation to abdomen, and ⅛ gr. morphia every hour.

6 P. M. Bowels moved; vomiting continues; matters thrown up of a bilious character; skin cold, and wet with perspiration; pulse feeble and quick. Ordered brandy to be freely administered and hot bricks to be applied to feet.

26th, 9 A. M. Patient excessively prostrated, and still vomiting the greenish looking matter. Pulse barely perceptible; respiration labored.

Died at 3 P. M.

*Autopsy.*—On opening the cavity of the abdomen found an effusion of serum, lymph and pus; recent adhesions and the intestines distended with gas. The whole peritoneal sac was injected, presenting an arborescent appearance. The cavity of the uterus was of a dark gangrenous hue and fetid odor. The gall bladder was full, and there was an effusion of serum into the cavity of the pleura and pericardium.

*Case* III.—Elizabeth Riley, aged 35, native of Ireland, temperate and of good constiution, was delivered of her fourth child, January 30th, 8 P. M., the labor being easy, and lasting fourteen hours.

31st; Very comfortable. No motion from bowels since delivery. Gave castor oil, one and a half ounces, which operated four times.

Feb. 1st, 2 A. M. Complains of slight pain in the hypogastric region, and says she had a light chill the afternoon previous. The secretion of milk is small; lochial discharge free; pulse small and wiry, beating 140 a minute; besides she is hysterical. Sat patient up in bed and bled her to approaching syncope, taking about 30 ounces, during which the pulse fell to one hundred and became full and more natural. Ordered one grain of calomel and half a grain of opium every hour.

9 A. M. Feels better; rested pretty well after bleeding, previous to which she had slept but little. The pain in the hypogastric region is not so intense; skin dry and tongue coated white; pulse one hundred and twenty and small. Gave her ten grains of calomel, with five of camphor, and one of opium. Ordered an injection of one ounce of assafetida, and one grain of calomel, with one of opium every hour. Also ordered a turpentine strap to abdomen.

8 P. M. Says she feels much better; has had a comfortable sleep of two hours duration; skin moist; pulse one hundred and fifteen, and full; some distention and tenderness of abdomen. Ate a little gruel during the day. Continue last prescription.

12 o'clock. Sleeping soundly; respiration twenty in a minute, and laborious; pulse full; has had four dark colored alvine dejections.

Feb. 2d, 9 A. M. Slept comfortably most of the night, and feels pleasantly to-day. But little pain over abdomen on pressure; lochia flowing well; pulse one hundred and eighteen, and full; had one dark colored discharge; complains of thirst; tongue in better condition. Ordered pil. calomel comp. and opium, of each a grain, every hour.

2 P. M. Pretty easy; no pain in abdomen; pulse one hundred and twenty in a minute, not so full; appetite good.

At 12 o'clock she is comfortable.

3d, 9 A. M. Slept most the night; pulse one hundred; appetite good. Continue same treatment.

2 P. M. Improving. Continue same prescription, with half the quantity of opium.

In the evening she is feeling pretty well; appetite tolerably good, and has had one natural discharge from her bowels.

4th. Rested badly last night; bowels moved six or eight times, and she feels very weak. Ordered brandy punch, and to continue calomel and opium every two hours.

5th. Rested badly, and had two discharges from bowels. Has a cough which causes pain in the abdomen.—Ordered to stop last prescription.

6th. Very weak, and bowels opened once since last date. Found a mucous rattle over both lungs; feeble respiration and crepitous at the base of right lung; pulse one hundred and thirty, and small; tongue coated; has no pain. Ordered calomel gr. j. and opium half a grain, every two hours. Also mist. expector. and vin. antimony.

7th; Feels pretty smart, though she rested badly; cough is easier, and bowels have moved twice this morning.

This evening she has no pain anywhere, but is quite weak, having had four alvine evacuations and vomited several times. Ordered weak brandy punch.

8th. Bowels moved twice last night, and she slept but little. Pulse continues frequent and pretty strong. Suspended medication and ordered nourishing diet.

9th. Bowels still loose. Feels no pain; expectorates none; the mucous rattle is audible several feet from the bed. Ordered an enema of tr. opii ʒj. and starch q. s., which is to be repeated after every second discharge.

10th. Slept but little last night. Bowels were twice moved; tongue somewhat enlarged and purple. She vomits a greenish matter whenever the pain is severe; pulse one hundred and thirty, and full; mucous rattle as before. Ordered tart. antimony, the eighth part of a grain every two hours.

At 8 o'clock, P. M., saw her again. Has expectorated a little mucous tinged with blood; breathing is difficult and pulse feeble; heard a mucous rattle in left lung and the apex of right, with bronchial respiration in inferior two-thirds of right lung. Ordered aqua camphor. ℥ iv., ammonia carb. ℈j., of which a tablespoonful every hour; sinapisms to chest.

This woman died early on the morning of the 11th.

*Autopsical appearances.*—The abdomen was filled with pus; and the pus was gathered, also, in sacs, formed by lymph, in the convolutions of the intestines. The bowels were strongly agglutinated together. There was no effusion of serum, at least it was not discoverable. The uterus was healthy. There was effusion of serum into the pleural cavity; bronchitis in both lungs, and pneumonia in the inferior portion of the right lung.

It is remarkable in this case that the pain in the abdomen ceased some days before death.

Before closing this report, I trust I may be allowed a few words respecting the Hospital and our efficient head, Dr. Reese. He has proved himself highly fitted for the post he now occupies—a man filled with the desire to benefit the patients and the profession. Under his fostering care, the Hospital has come to be one of the first institutions of the kind in this country, instead, as it formerly was, a shame to this great city.

Bellevue Hospital, June, 1849.

